# Impacts of Formula Supplemented with Milk Fat Globule Membrane on the Neurolipidome of Brain Regions of Piglets

**DOI:** 10.3390/metabo12080689

**Published:** 2022-07-26

**Authors:** Karl Fraser, Leigh Ryan, Ryan N. Dilger, Kelly Dunstan, Kelly Armstrong, Jason Peters, Hedley Stirrat, Neill Haggerty, Alastair K. H. MacGibbon, James Dekker, Wayne Young, Nicole C. Roy

**Affiliations:** 1 AgResearch Ltd., Grasslands Research Centre, Palmerston North 4442, New Zealand; leighjoyceryan@gmail.com (L.R.); kelly.dunstan@fonterra.com (K.D.); kelly.armstrong@esr.cri.nz (K.A.); jason.peters@agresearch.co.nz (J.P.); contact@heds.nz (H.S.); wayne.young@agresearch.co.nz (W.Y.); nicole.roy@otago.ac.nz (N.C.R.); 2 Riddet Institute, Massey University, Palmerston North 4442, New Zealand; 3 High-Value Nutrition National Science Challenge, Auckland 7039, New Zealand; 4 Department of Animal Sciences, University of Illinois, Urbana, IL 61820, USA; rdilger2@illinois.edu; 5 Fonterra Research and Development Centre, Dairy Farm Rd., Palmerston North 4442, New Zealand; neill.haggerty@fonterra.com (N.H.); alastair.macgibbon@fonterra.com (A.K.H.M.); james.dekker@fonterra.com (J.D.); 6 Department of Human Nutrition, University of Otago, Dunedin 9016, New Zealand

**Keywords:** infant formula, milk fat globule membrane, piglets, brain, lipidome, cerebellum, hippocampus, prefrontal cortex, neonatal, metabolomics

## Abstract

The milk fat globule membrane (MFGM) appears to play an important role in infant neurocognitive development; however, its mechanism(s) of action remains unclear. This study aimed to investigate the role of a dietary MFGM supplement on the lipid profiles of different neonatal brain regions. Ten-day-old male piglets (4–5 kg) were fed unsupplemented infant formula (control, n = 7) or an infant formula supplemented with low (4%) or high (8%) levels of MFGM (n = 8 each) daily for 21 days. Piglets were then euthanized, and brain tissues were sectioned. Untargeted liquid chromatography-mass spectrometry lipidomics was performed on the cerebellum, hippocampus, prefrontal cortex, and the rest of the brain. The analyses identified 271 and 171 lipids using positive and negative ionization modes, respectively, spanning 16 different lipid classes. MFGM consumption did not significantly alter the lipidome in most brain regions, regardless of dose, compared to the control infant formula. However, 16 triacylglyceride species were increased in the hippocampus (t-test, *p*-value < 0.05) of the high-supplemented piglets. Most lipids (262 (96.7%) and 160 (93.6%), respectively) differed significantly between different brain regions (ANOVA, false discovery rate corrected *p*-value < 0.05) independent of diet. Thus, this study highlighted that dietary MFGM altered lipid abundance in the hippocampus and detected large differences in lipid profiles between neonatal piglet brain regions.

## 1. Introduction

The brain is a heterogeneous organ and a critical part of the central nervous system. It controls not only the essential bodily functions such as breathing and other metabolic functions but is also responsible for learning and memory, behavior, and thoughts [1]. The brain is the second most lipid-rich organ in the mammalian body [2] and is composed of numerous lipid classes, such as phospholipids, sphingolipids, ceramides and triacylglycerides (TG) [3]. These lipids have important functions, including cellular signaling, forming the lipid bilayers of cellular membranes, and energy storage [4]. Its complex structures and functions are also reflected by the many lipid species detected [1,5,6,7]. However, the regulation of the brain lipidome and its functions in metabolism and signaling are poorly understood.

Dietary consumption of lipids has been implicated in brain development, with a recent study showing increases in mRNA expression of genes involved in brain function as a response to milk fat globule membrane (MFGM) consumption [8]. In addition, feeding mice diets that differed in fatty acid saturation degree (beef tallow vs. safflower vs. fish oil) resulted in minor differences in the brain lipidome across multiple brain regions, especially for glycerophospholipid species. In contrast, diets enriched in n-3 fatty acids, eicosapentaenoic acid (EPA) and docosahexaenoic acid (DHA) (fish oil) altered the phospholipid fatty acid composition [5]. In addition, long-term saturated fat intake has been shown to impact brain lipid composition in mice, particularly leading to perturbations in individual phospholipid species [6]. Infant formulas and milk replacers enriched in DHA and arachidonic acid (ARA) fed to growing piglets resulted in increased incorporation of DHA and ARA into brain tissue, and this increase was linked to behavioral observations, particularly around sleep and general activity levels [9,10].

The MFGM is part of the lipid delivery mechanism of milk, whereby triglyceride droplets are encased in a phospholipid-rich tri-layer membrane that also contains other glycosylated lipids, proteins and lipid-soluble vitamins [11]. Complex lipids (e.g., gangliosides, sphingolipids and other forms of polar phospholipids) that are naturally present in mammalian milk are known to be beneficial for gastrointestinal development and maturation [12], brain development, cognition and behavior [13,14] in human infants.

Despite the benefits of MFGM, low-fat dairy products (e.g., skim milk) are typically used in infant formulas because bovine milk contains a higher proportion of undesirable saturated fatty acids than human milk [15]. As the MFGM portion has generally been discarded during infant formula manufacturing, its levels in traditional standard infant formula are lower than those found in human milk [16,17]. However, there has been increasing interest in the properties of MFGM. For example, studies showed that when infant formulas made with bovine milk were supplemented with bovine MFGM, improved neurological development measures in infants compared to infants fed unsupplemented bovine infant formula were observed [13,18,19]. Changes in neurodevelopmental structure have also been observed in piglets fed formula supplemented with ingredients such as a mix of prebiotics, lactoferrin and MFGM [20]. In contrast, a recent study of formula supplemented with MFGM alone reported the formula was well tolerated, but there were no changes in brain cholesterol concentrations, macrostructure, microstructure, or recognition memory in the piglets [21]. Thus, considerable interest remains in understanding the effects and potential benefits of enriching infant formula with MFGM for infant growth and performance [11].

The present study aimed to determine if dietary supplementation of an infant formulation with MFGM at two different doses (comparable to the lower (4% MFGM) and higher (8% MFGM) levels of MFGM found in human milk [22]) could influence the lipid composition of specific brain regions, or of plasma, in piglets as a model of human infants. Piglets share similarities in gastrointestinal tract physiology and gross neuroanatomy to humans. These features make the piglet an excellent model to investigate the impact of dietary changes on brain function and development. Brain lipid profiles of the cerebellum, hippocampus, prefrontal cortex, and rest-of-brain samples, along with plasma lipidome and polar metabolite profiles, were measured using positive and negative ionization mode untargeted liquid chromatography-mass spectrometry (LC-MS).

## 2. Results

### 2.1. Brain Region Lipidome

#### 2.1.1. Lipid Annotation and Identification

The LC-MS analysis of brain extracts revealed 271 annotated lipid species from positive mode ionization analyses spanning 13 lipid classes and 171 annotated lipid species from negative mode ionization analyses spanning 14 lipid classes. In total, this annotation covered 16 lipid classes, given that some lipid classes were detected in both ionization modes, e.g., phosphatidylcholine (PC), while other lipid classes only ionized in a single mode, e.g., TG species only ionized in positive ionization mode.

Major classes measured (and number of individual species summed across both ionization modes) included phosphatidylethanolamines (PE) (104), (PC) (73), phosphatidylserines (PS) (58), sphingomyelins (SM) (43), TG (37), diacylglycerides (DG) (30), sterol esters (ST) (27), monoglycosylceramides (CerG1) (23), phosphatidylglycerols (PG) (14), ceramides (Cer) (10), and phosphatidylinositols (PI) (9). A full list of annotated lipids identified by MS^2^ matching spectral library using LipidSearch and MS^1^ accurate mass matching using LipidBlast, matched with the XCMS results table for each ionization mode, is provided in Table S1. This approach yielded 163 of the 271 annotated lipids in positive ionization (~60%) matched using MS^2^ spectral data, while 81 of the 171 annotated lipids in negative ionization mode (~47%) were matched using MS^2^ spectral data.

#### 2.1.2. Lipid Class Composition

Each lipid species was summed together based on lipid class to examine differences in brain composition across the four regions sampled at the lipid class level regardless of formula intervention. A lipid class total abundance for the 23 sub-samples of each brain region was created for each ionization mode.

**Figure 1:**
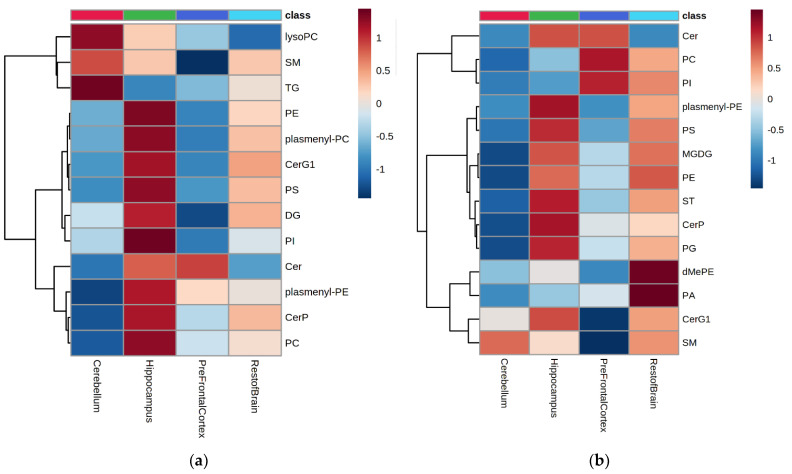
Heatmap of mean total lipid abundances at the lipid class level for four brain regions (cerebellum, hippocampus, prefrontal cortex, and the rest of the brain) in positive mode (**a**) and negative mode (**b**), regardless of intervention (n = 23 animals). Average of sum peak area data log10 normalized and auto-scaled for each lipid class, red = high abundance and blue = low abundance levels. Cer = ceramide, CerG1 = monoglycosylceramide, CerP = ceramide phosphate, DG = diacylglyceride, lysoPC = lysophosphatidylcholine, PA = phosphatidic acid, PC = phosphatidylcholine, PE = phosphatidylethanolamine, PG = phosphatidylglycerol, PI = phosphatidylinositol, PS = phosphatidylserine, SM = sphingomyelin, ST = sterol ester, TG = triacylglyceride.

Statistical analysis of these two datasets using ANOVA highlighted that all 13 species and 13 out of 14 classes in positive and negative ionization modes were significantly different across the brain tissues (*p* < 0.05). Figure 1 highlights the lipid profile across the brain regions as detected by each ionization mode (based on log10 normalized and auto-scaled intensities of each class).

**Figure 2:**
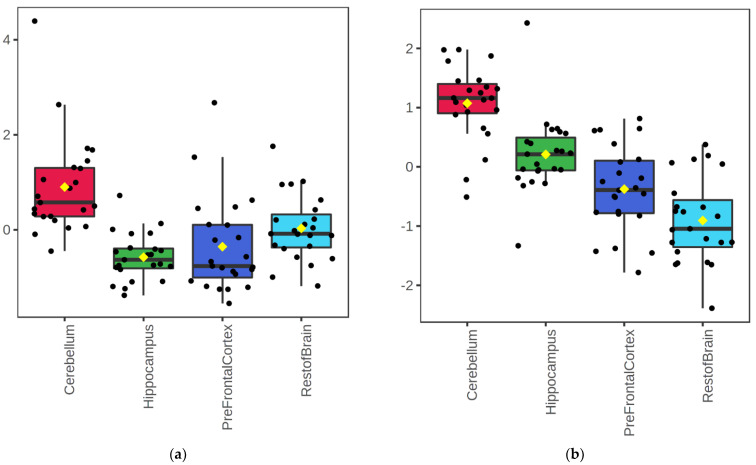

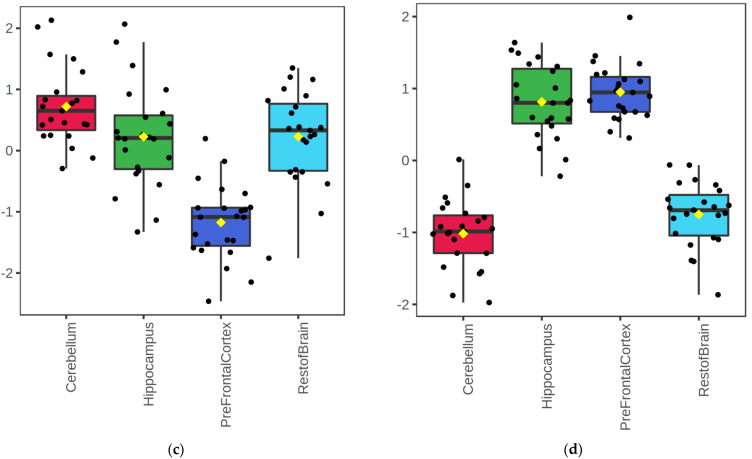
Boxplots of total lipid abundance at the lipid class level for four brain regions (cerebellum, hippocampus, prefrontal cortex, and the rest of the brain) in positive mode for triacylglycerides (**a**), lysophosphatidylcholines (**b**), sphingomyelin (**c**), and ceramides (**d**). Data shown are the sum of all peak areas per lipid class, log10 normalized and auto-scaled.

In the brain regions measured, most of the phospholipid classes (PC, PE, PS, PI, PG, plasmenyl-PC, and plasmenyl-PE) were highest in abundance in the hippocampus, except for the PC and PI species detected by negative ionization, which were higher in the pre-frontal cortex (Figure 1). In contrast, TG and lysophosphatidylcholines (lysoPC) were highly abundant in the cerebellum (Figure 1a and Figure 2a,b). In addition, the relative abundance of SM was lower in the prefrontal cortex (both ionization modes) than in the other tissues measured here (Figure 1a,b and Figure 2c). Finally, Cer, CerG1 and ceramide phosphates (CerP) were highest in abundance in the hippocampus compared to the other areas (Figure 2d).

The mean abundances of each lipid species were calculated for each brain region to understand the relative brain composition at the lipid class level. These mean abundances were then summed per lipid class, and a percentage was calculated for each lipid class per ionization mode. Across the four brain regions sampled, PC, PE and SM were the predominant lipid classes, constituting on average approximately 73.6%, 9.7% and 8.2%, respectively, of all lipids detected in positive ionization mode. The remaining approximately 8.5% was made up of Cer, DG, TG, and other minor abundant phospholipid species. In negative mode, the phospholipids again dominated the percentage composition measured with PE, PS and PC, constituting approximately 48.5%, 19.7% and 13.5%, respectively. The remaining accounted for approximately 18.3% and were SM, Cer, sterols, and other minor phospholipid classes. Full results are provided in Table S2.

Given that summing the lipid classes to a single number reduces the detailed species resolution of the hundreds of lipids in the lipidome measured using LC-MS, the data were subsequently analyzed at the individual lipid species level.

#### 2.1.3. Lipid Species Composition

**Figure 3:**
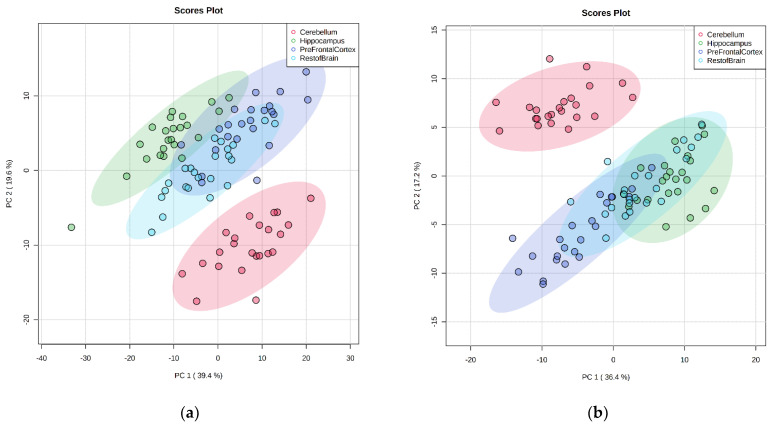
Principal component analysis score plot of four brain regions (cerebellum, hippocampus, prefrontal cortex, and the rest of the brain) in positive mode (**a**) and negative mode (**b**), regardless of intervention (n = 23 animals).

Multivariate analysis of the individual lipid species identified using principal component analysis (PCA) also highlighted large differences in lipid composition according to brain region (Figure 3). Notably, the cerebellum resolved furthest away from the other regions in both ionization modes in the two-dimension (2-D) PCA score plot. The hippocampus also resolved away from the prefrontal cortex and the rest of the brain. In addition, the PCA score plots captured over half of the variation in the sample set in the first 2-D (59% and 53.6% for positive and negative ionization, respectively). Principal component 1 (PC1) captured 39.4% and 36.4 %, respectively, of the variation, and principal component 2 (PC2) contributed 19.6% and 17.2%, respectively, of the variation in the positive and negative ionization modes.

**Figure 4:**
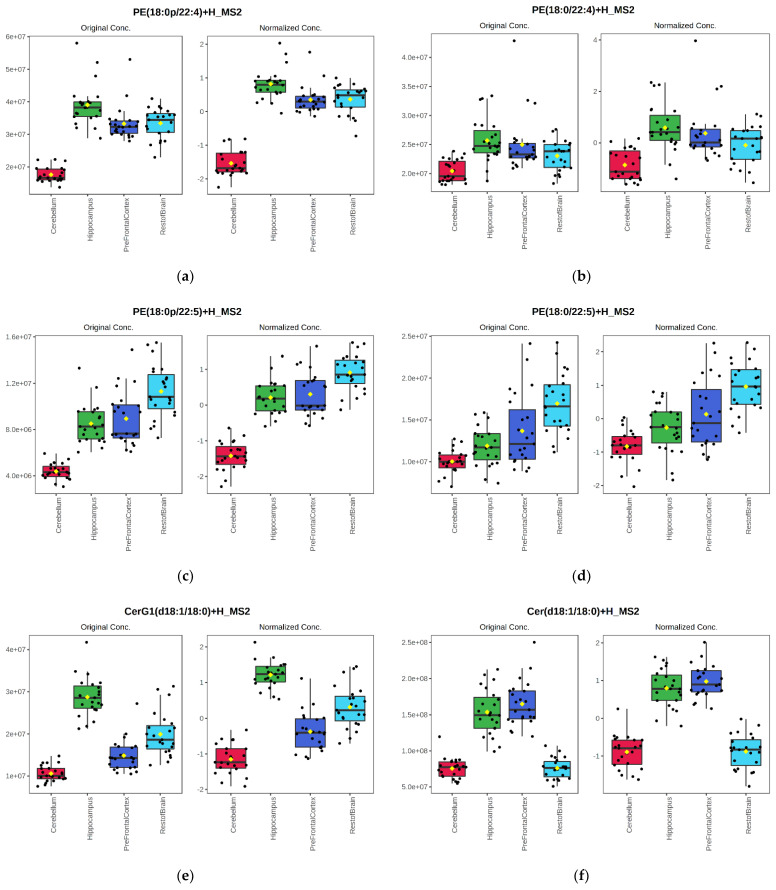
Peak area bar charts and log10 transformed and auto-scaled peak area boxplots of six lipid species detected across the four brain regions (cerebellum, hippocampus, prefrontal cortex, and the rest of the brain) in positive mode, regardless of intervention (n = 23 animals). Lipid IDs for (**a**–**f**) are given in the boxplot figure legends. All six lipid species shown were significantly different across brain regions (FDR corrected *p* < 0.05) by ANOVA.

The univariate analysis revealed that 262 of the 271 (96.7%) lipid species detected in positive ionization mode and 160 of 171 (93.6%) lipid species detected in negative ionization were significantly different (Fisher’s LSD test, FDR adjusted *p*-value < 0.05) across the four brain regions. Lipidomic analysis revealed significant lipid species-level differences related to both brain region structure and function. For instance, plasmenyl-PE levels of two similar fatty acid-containing species (Figure 4a, PE(18:0p/22:4) and Figure 4c, PE(18:0p/22:5)) followed similar distribution patterns. The lowest levels of these lipids were observed in the cerebellum, whereas the hippocampus, prefrontal cortex and the rest of the brain had similar levels. Their respective PE species (Figure 4b,d) show a similar pattern. However, the differences between the cerebellum and the other tissues were much smaller than for their respective plasmenyl-PEs. Notably, the relative concentration of both 22:5-containing fatty acid PE species in the rest of the brain sample was higher than in the other three tissues (Figure 4c,d), suggesting the region-specific distribution of different fatty acids across the brain.

Another example of tissue distribution differences was Cer(d18/18:0) and glucosylceramide(d18/18:0) (Figure 4e,f). The Cer(d18/18:0) species (Figure 4f) followed the same pattern observed for the Cer class, i.e., highest in the hippocampus and prefrontal cortex (Figure 1 and Figure 2) and lower abundance in the cerebellum and prefrontal cortex. However, the glucosylceramide containing the same fatty acid [glucosylceramide(d18/18:0)] (Figure 4e) had a similar high abundance in the prefrontal cortex and the hippocampus.

### 2.2. Dietary Intervention Impact on Brain Lipidome

#### 2.2.1. Lipid Class-Level Analysis

The two-way ANOVA analyses revealed no effects of low- or high-dose MFGM supplementation of infant formula compared to the control formula on brain lipidome abundance at the class level. This was observed across all four regions or in each brain region. There was also no effect when comparing the low dose to the high dose of MFGM. These results hold for either ionization mode (data not shown).

#### 2.2.2. Lipid Species-Level Analysis

As was the case for class-level analysis, the two-way ANOVA analyses failed to detect any impact of the infant formula supplemented with low or high MFGM dose compared to the control infant formula on individual lipid species abundances across the four brain regions or within each brain region. There was also no effect when comparing the low dose to the high dose of MFGM. These results hold for either ionization mode (data not shown).

An alternative approach was then employed to determine if more minor but combinatorial differences in the lipid species data could be detected by partial least squares-discriminant analysis (PLS-DA). This method creates a model separating the control infant formula, the low-MFGM infant formula, and the high-MFGM infant formula for each of the four brain regions. Then, each of these models underwent feature selection and filtering by selecting all features in the model with a variable importance in projection (VIP) score superior to 1.0 to create a new model. These four new VIP filtered models (one for each brain region) were then tested via permutation calculations (200) and cross-validation ANOVA (CV-ANOVA) methods to check for model validity. These VIP-filtered and optimized models generated were not significant (*p* > 0.05) or robust, with all four models having Q2 values < 0.1.

A comparison of the high-dose formula to the control formula was subsequently performed. The univariate analysis of the lipid abundance for each brain region was carried out by t-test to compare the high dose and control groups. None of the four brain areas had significantly different lipids after FDR correction in either ionization mode. For the hippocampus region, 30 lipids in positive ionization mode and 2 lipids in negative ionization were lower in abundance with the higher MFGM dose at an unadjusted *p*-value < 0.05. The rest of the brain (2 in positive mode, 3 in negative mode), cerebellum (1 in positive mode, 1 in negative mode), and prefrontal cortex (1 in positive mode, 2 in negative mode) showed few significant differences with an unadjusted *p*-value < 0.05. Thus, the differences observed outside the hippocampus were likely to be random and not considered further and are consistent with the hippocampus being a major location of many lipid species, as shown in Figure 1.

The changes in lipid abundance in the hippocampus are listed in Table 1. Notably, of the 30 lipids, 16 were TGs, and 29 were polyunsaturated. The TGs were less abundant in the hippocampus of the piglets fed the higher dose of MFGM infant formula than those fed the control formula. In contrast, 6 PCs, 3 PEs, 1 lysoPC, 1 plasmenyl-PC, 1 plasmenyl-PE, 1 Cer, and 1 glucosylceramide had a higher abundance in the hippocampus of the piglets fed the higher dose of MFGM infant formula than those fed the control formula. Boxplots of the abundance of all 30 lipids listed in Table 1 are provided in Supplementary Figure S1.

Finally, a multivariate approach was performed using the two-group comparison described above between the piglet fed the higher MFGM infant formula and the control infant formula. The PLS-DA models were created for each brain region, and model optimization using feature selection (via VIP filtering) was again performed.

**Figure 5:**
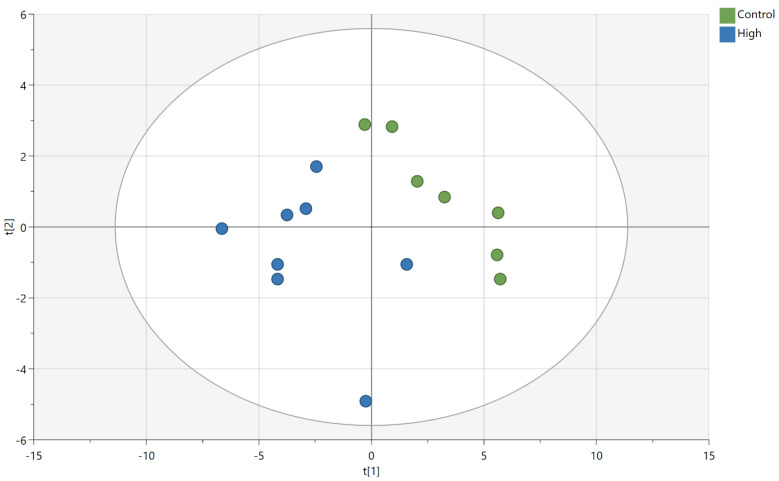
PLS-DA plot of lipids measured in the hippocampus of pigs fed the high-MFGM supplemented infant formula or control formula in positive ionization mode, (n = 15 animals) Q2(cum) = 0.78. Control is the unsupplemented (control) infant formula; High is the highest level of the MFGM supplemented in infant formula.

The models for the cerebellum, prefrontal cortex and rest of the brain were not valid or significant. In contrast, the model for the hippocampus was significant (*p* = 0.018) and had a Q2(cum) of 0.78, suggesting the model was robust (Figure 5). The PLS-DA loading plot (Supplementary Figure S2) for the selected features gave similar results to the univariate approach, with a range of phospholipid species and a glucosylceramide linked to the MFGM supplemented infant formula and the TG linked to the control samples. Of the 25 lipids selected using VIP filtering to build the refined PLS-DA model (Figure 5), 22 of them were listed as significant features (Table 1; Supplementary Figure S3).

#### 2.2.3. Plasma Metabolomic and Lipidomic Analyses

The LC-MS metabolomic analysis of polar metabolites detected in the plasma extracts revealed 152 and 131 metabolite features from positive and negative mode ionization analyses, respectively. The LC-MS lipidomic analysis of the plasma extracts detected 308 and 580 lipid features from positive and negative mode ionization analyses, respectively. Prior to conducting the feature annotation steps, univariate and multivariate statistical analyses were performed as described for the brain extracts above (including comparing only high-MFGM supplemented infant formula vs. the control infant formula). These analyses revealed that none of the metabolite or lipid feature abundances were significantly impacted by the infant formula supplementation.

## 3. Discussion

This study reports the impacts of consuming an MFGM-supplemented IF for 21 days on the brain lipidome, plasma lipidome and polar metabolite profile of growing piglets. This study also reports a detailed lipidome profile of multiple regions of the piglet brain for the first time.

The lipid annotation of the brain tissues detected 271 and 171 lipid species in positive and negative ionization modes, respectively, across the four brain regions. The most abundant lipid species were the phospholipid classes, predominantly PC and PE. Phospholipids are the most abundant lipid class within the brain and perform many functions beyond membrane structure. For example, they can act as a storage pool for fatty acids used in cellular signaling and contribute to cellular membrane potential [23]. A previous LC-MS analysis of the whole porcine brain identified approximately 160 lipid species spanning 13 lipid classes [24]. The authors used a similar Folsch-type extraction method but a different chromatography method (HILIC) that did not detect the TG portions of the brain extracts. Their results at the class level were also in accordance with the results reported here and with other studies investigating brain lipid composition [1,5,25].

Another key difference compared to other studies is that the brain was dissected into different spatial regions for subsequent analysis, partly similar to the method used in studies in mice [1,4,5,6]. This approach highlighted the differences in lipid class and individual lipid species between the porcine brain regions. For example, the cerebellum contained substantially higher levels of TG compared to the other regions measured. Compared to the other lipid classes, TGs play a small role in neuronal lipid metabolism. However, they do act as the storage form of lipid precursors [23]. Our findings suggest that the cerebellum, while thought to be important for motor control and possibly cognition in humans, may also act as a storage region for some precursors of lipid metabolism. Areas rich in particular species also included the hippocampus and prefrontal cortex, in which Cer was highly abundant, a phenomenon observed in adult mice [5]. Another example was SM being lower in the prefrontal cortex compared to the other tissues measured. While this finding may reflect how the different regions of the brain function, its exact significance remains to be elucidated.

Our results identified variation in individual lipid species abundances and changes in distribution in lipids of the same class but containing different fatty acids across the brain regions. Some of the changes noted included the change in distribution across the tissues between the PEs and plasmenyl-PEs. Plasmenyl-PEs are known to play a critical role in myelin sheath formation and function, and PEs are known to be important in cell membrane function. The differences in these two compounds across tissue types likely reflect that the cerebellum contains less myelin and cell membrane-related lipids. This variation across different brain tissue types has been observed in studies investigating the incorporation of DHA in young mice and rats fed infant formula enriched with DHA [26,27]. Although the consequences of these species-level differences are difficult to interpret biologically, they nevertheless highlight the value of measuring the extra level of lipidome from different brain regions to ensure sample measurements are comparable when investigating possible dietary intervention effects compared to simple total class level measurements.

Studies on commercial MFGM products have shown compositional consistency across the MFGM lipidome, particularly for the lipid species detected. While there are minor differences in lipid abundance within different MFGM ingredient preparations, generally, MFGM products contain over 300 lipid species [28]. TGs are the most abundant lipid class in MFGM ingredients, followed by PC and SM, and the phospholipids are thought to be at biologically significant levels [29].

Regardless of dose, none of the lipid species were significantly different (after FDR correction) after consuming MFGM-supplemented infant formula for 21 days in any of the four brain regions measured. However, 30 lipids were shown to be different in the hippocampus using less stringent tests (univariate and multivariate PLS-DA) when comparing the high-dose infant formula to the control formula. These findings indicate that MFGM supplementation may impact the physiology of the brain, but high variation between piglets might obscure the effect. Furthermore, of the 30 lipids with altered levels in response to high-dose MFGM formula, 16 were TGs that were all lower in abundance in the hippocampus samples of the piglets fed the higher dose of MFGM infant formula compared to the piglets fed the control formula. Previous studies in piglets fed a mixed lactoferrin/MFGM/oligosaccharide formula have shown changes in brain microvascular structure [20], while another study with an MFGM-only supplemented formula detected no differences in brain cholesterol or brain macrostructure and microstructure [21]. However, no lipidomic analysis of the brain was done in these studies. Notably, from the 37 TG detected across all brain regions, 16 (43%) were significantly different in the hippocampus due to treatment, and all were trending in the same direction, i.e., all decreased in MFGM infant formula-fed piglets. Trends observed across whole lipid classes such as this support the occurrence of brain tissue molecular changes related to formula with MFGM consumption at the higher dose. Likewise, six PCs were increased in the hippocampus extracts of the MFGM-fed piglets. One possible explanation for this is the digestion of choline-containing phospholipids (higher in abundance in the MFGM supplemented infant formula) and subsequent transport of choline to the brain. Choline is crucial for brain function and serves as the precursor to the biosynthesis of PC [30]. The digestion and subsequent uptake of the choline-containing classes (PC, SM) are regulated by several factors. PC is digested independent of choline, while SM is absorbed intact, and choline is absorbed from the duodenum, jejunum, and ileum [30]. Several studies in mice have shown increases in phospholipids in multiple regions of the mouse brain in response to modified fat diets [6,27,31]. Thus, the higher concentrations of PC lipid species in piglets supplemented with the higher dose of MFGM infant formula may be linked to higher levels of PC in the supplemented infant formula or altered digestion/metabolism effects. This finding may also be the case for the glucosylceramide elevated in the hippocampus from the piglets fed the higher dose of MFGM infant formula. Glucosylceramides have been observed at detectable concentrations in MFGM products [28].

This study has shown the importance of spatial lipidomics (i.e., lipidomics analyses of different brain regions). However, one of the limitations of this approach is the difficulty in some brain regions to visually define the different tissues during dissection, possibly leading to both blending signals and diluting of regional differences due to diet. It is also possible that the subtle differences due to the addition of MFGM may occur in low-abundance lipids. A targeted lipidomic approach may potentially better detect differences in minor lipids, particular classes of lipids, or targeted individual lipid species not measured using this untargeted approach.

Overall, this study has shown large differences in brain lipid composition across multiple regions of a growing porcine brain and provided evidence that consumption of infant formula supplemented with a high dose of MFGM for 21 days can alter the lipid composition of the hippocampus in piglets. Higher doses or longer feeding of MFGM or altered formulations that contain higher levels of phospholipid-related moieties may be required to observe greater changes in brain tissue lipid structure and composition. The possible incorporation of dietary lipids into brain tissue could be explored using stable isotope experiments, combined with spatial tracking of dietary lipids to the different regions of the brain.

## 4. Materials and Methods

### 4.1. Animal Trial and Sampling

This study was carried out in strict accordance with the New Zealand Animal Welfare Act 1999 and was approved by the AgResearch Limited (Grasslands) Animal Ethics Committee (Ethics Approval No.: 13947).

Twenty-four male large white cross ten-day-old piglets were obtained from a commercial farm in the Manawatu-Wanganui region of New Zealand. All piglets were housed in custom cages constructed to allow animals to see, hear, and smell adjacent piglets but still prevent physical contact [32]. On arrival at the animal facility (day 1), the piglets were pair-housed for two nights. The piglets were exclusively fed reconstituted infant formula (control formula) two hours post-arrival and then every four hours after that feed. From days 2 to 24, the piglets were individually housed. During this period, the piglets were let out into a shared pen and allowed to interact for an hour of social time each day.

From days 3 to 24, the piglets were assigned to one of three treatment groups; eight receiving a control formula, eight receiving a control formula supplemented with 4% NZMP MFGM Lipid 100 (Low), and eight receiving a control formula supplemented with 8% NZMP MFGM Lipid 100 (High). All formulas were dispensed using an automated system programmed to offer the formula every two hours with automatic measurement of refusals. The piglet caging and automated feeding system was designed and manufactured by ShapeMaster (Ogden, IL, USA).

On day 24, 23 piglets were euthanized by sedation using a cocktail of tiletamine hydrochloride, zolazepam hydrochloride, xylazine, and ketamine hydrochloride (final solution 50 mg/mL of each drug), followed by intracardiac puncture with sodium pentobarbitone (300 mg/mL). One control piglet was euthanized during the study due to ill health. The brain was dissected into four regions: the left and right prefrontal cortex (behavior, motor skills, and problem-solving), cerebellum (balance and coordination), hippocampus (memory formation, memory organization, and memory storing), and a rest-of-brain portion. All brain tissue samples were stored at –80 °C until analysis. Blood samples were collected via cardiac puncture (approximately 20 mL) immediately after sedation (approximately 10 min later) with an EDTA-coated syringe and needle, put into a 50 mL Falcon tube, and spun at 2000× *g* for 10 min at room temperature. An aliquot of plasma was removed and stored at –80 °C until analysis.

### 4.2. Brain and Plasma Extractions

The brain samples were thawed on ice. Portions from the left and right prefrontal cortex, the cerebellum and the hippocampus were taken into 2 mL Eppendorf tubes, while half of the rest-of-brain portion was taken into a 70 mL plastic bottle. All samples were homogenized (30 Hz, 2 × 30 s) with either a single bead per tube (2 mL Eppendorf tubes) or four beads (70 mL pottles) using a tissue lyzer (QIAGEN TissueLyser II, QIAGEN, Hilden, Germany). The samples were weighed into fresh Eppendorf tubes. First, to 50 ± 5 mg of brain tissue was added 800 µL of CHCl_3_:MeOH (1:1 *v*/*v*, stored at –20 °C), and the sample was homogenized (30 Hz, 2 × 30 s). Next, the sample was diluted with 400 µL of water, vortexed (30 s) and centrifuged (4 °C, 18,213 rcf, 10 min). Finally, 200 µL of the lower organic layer was taken and dried under a stream of N_2_. For LC-MS analysis, the dried extract was redissolved in 300 µL of modified Folsch solution (CHCl_3_:MeOH:H_2_O, 66:33:1 *v*/*v*/*v*, containing 0.01% *w*/*v* 16:0 d_31_-18:1-PE internal standard) and then diluted a further ten-fold, vortexed (30 s), and 150 μL was transferred into a glass insert in a sample vial for lipid analysis, while 20 μL was transferred into an Eppendorf for the pooled quality-control sample.

Similarly, plasma samples were thawed on ice, and a 100 μL aliquot was extracted using the bi-phasic extraction for lipidomics (organic fraction) and metabolomics (polar fraction) described above and previously reported elsewhere [33]. For LC-MS lipidomic analysis, the dried extract was redissolved in 200 µL of modified Folsch solution (CHCl_3_:MeOH:H_2_O, 66:33:1 *v*/*v*/*v*, containing 0.01% *w*/*v* 16:0 d_31_-18:1-PE internal standard) and 150 μL was transferred into a glass insert in a sample vial for lipid analysis, while 20 μL was transferred into an Eppendorf for the pooled quality-control sample. For LC-MS metabolomic analysis, the dried extract was redissolved in 200 μL acetonitrile:H_2_O (50:50, *v*/*v*) and 150 μL was transferred into a glass insert in a sample vial for metabolomic analysis, while 20 μL was transferred into an Eppendorf for the pooled quality-control sample.

### 4.3. LC-MS Lipidomics

The analyses of brain and plasma extracts, QC samples and solvent blanks were performed as previously reported [34]. Briefly, the analysis of the lipidome was performed using a Thermo Q-Exactive LC-MS system spectrometer (Thermo Fisher Scientific, San Jose, CA, USA) using positive and negative electrospray ionization (source operated at 300 °C) and the data collected from *m/z* 200–2000 for the MS^1^ spectra. The capillary temperature was 300 °C. Aliquots (2 µL) of extract were injected into a CSH C18 UHPLC column (1.7 µm particle size, 2.1 × 100 mm) held at 65 °C and eluted over a 17 min gradient with a flow rate of 600 μL min^−1^. The mobile phase was a mixture of isopropanol:acetonitrile (9:1 *v*/*v* with 0.1% formic acid and 10 mM ammonium formate) (solvent A) and acetonitrile:water (3:2 *v*/*v* with 0.1% formic acid and 10 mM ammonium formate) (solvent B). The gradient elution programme was as follows: 15–30% A (0–2 min), 30–48% A (2–2.5 min), 48–82% A (2.5–11 min), 82–99% A (11–11.5 min), held at 99% A (11.5–14 min), 99–15% A (14–14.1 min), held at 15% A (14.1–17 min).

The MS^1^ mass spectral data for quantifying the peak areas were collected at 35,000 resolution with a maximum trap fill time of 250 ms. For a subset of samples, the mass spectrometer was programmed to collect data-dependent MS^2^ fragmentation spectra on the most abundant ions in the MS^1^ scan with a normalized collision energy setting of 30. The MS^2^ spectral data used for identification with LipidSearch software (Thermo) were also collected at 35,000 resolution with a maximum trap fill time of 120 ms, and the isolation window of selected MS^1^ scans was ± 1.5 *m/z*. Positive ion mode parameters were as follows: spray voltage, 4.0 kV; capillary temperature, 275 °C; capillary voltage, 90 V, tube lens 120 V. Negative ion mode parameters were as follows: spray voltage, −2.5 kV; capillary temperature, 275 °C; capillary voltage, −90 V, tube lens −100 V. The nitrogen source gas desolvation settings were the same for both modes (arbitrary units): sheath gas, 40; auxiliary gas, 10; sweep gas, 5.

### 4.4. LC-MS Metabolomics

The analyses of plasma extracts, QC samples and solvent blanks were performed as previously reported [33]. Briefly, the analysis of the polar metabolite fraction was performed using a Thermo Exactive LC-MS system spectrometer (Thermo Fisher Scientific, San Jose, CA, USA) and positive and negative electrospray ionization (room temperature) and the data collected from *m/z* 55–11000. The capillary temperature was 325 °C, and the source voltage was set to 4.0 kV for both ionization modes. Aliquots were injected (2 µL) onto a SeQuant ZIC-pHILIC column (5 µm particle size, 2.1 × 100 mm) held at 25 °C and eluted over a 26 min gradient with a flow rate of 250 μL min^−1^. The mobile phase was A = 10 mM ammonium formate in H_2_O, B = 0.1% formic acid in acetonitrile. A gradient program was used at a flow rate of 250 μL min^−1^: 3–3% A (0.0–1.0 min), 3–30% A (1.0–12.0 min), 30–90% A (12.0– 14.5 min), and 90% A, which was maintained for 3.5 min followed by re-equilibration with 3% A for 7 min.

### 4.5. Data Extraction and Analysis

Peak areas were extracted from the samples by converting the Thermo raw mass spectrometry files to mzML using the MSConvert convert function of ProteoWizard [35] and then subsequently using the non-targeted peak detection tool XCMS [36] using in-house scripts in R (version 3.4.1) [37]. The resultant data matrix was then subjected to QC-based normalization (LOESS normalization) [38] and RSD filtering of normalized peaks in the QC samples (RSD < 0.3). Preliminary statistical data analysis (see below) was performed on the unannotated data matrices. Subsequently, the brain data matrix was matched with the lipid ions identified by LipidSearch (Thermo Fisher Scientific, San Jose, CA, USA) from the MS^2^ spectral data collected with a ±5 ppm mass error to annotate the lipid species. Subsequently, ions not matched by MS^2^ library searching (possibly due to lower intensity and therefore no MS^2^ data collected) were searched by accurate mass to the LipidBlast [39] (±0.008 Dalton mass error) and the hits were cross-referenced against the MS^2^ matches for class elution retention time to ensure any false annotations in the extra (on top of MS^2^ identifications) were minimized. Lipid annotations follow the standards reported based on more detailed LipidMaps terminology [40].

Statistical analysis was carried out on the resulting data matrix using MetaboAnalyst v5.0 [41] and SIMCA v16.0 (Umetrics, Umea, Sweden). Univariate analysis (ANOVA) and PCA of the brain regions was performed using MetaboAnalyst. In addition, SIMCA was used for the PLSDA of the infant formula interventions. All data underwent log_10_ transformation and autoscaling (mean-centred and divided by the standard deviation of each variable) before analysis.

## 5. Conclusions

The LCMS analysis of the brain lipidome showed that in growing piglets, the consumption of infant formula supplemented with a high dose of MFGM for 21 days impacted the hippocampal lipidome but did not appear to influence other brain tissues (cerebellum, prefrontal cortex, and rest of brain) that were assessed or the plasma. This finding indicates that MFGM supplementation of infant formula at the higher dose used here may alter the lipidome of some brain regions of the growing piglet. In addition, the analysis detected major differences in lipid profiles between the four different brain tissues measured in this study. This finding reinforces the importance of measuring individual brain regions to ensure correction for spatial resolution while examining for impacts of nutritional interventions.

## Figures and Tables

**Table 1:** Lipid species in the hippocampus detected using positive ionization mode in piglets fed the higher dose of MFGM infant formula compared to piglets fed the control infant formula. Note, annotations highlight the ionization adduct detected, and annotations ending with _MS^2^ are based on MS^2^ spectral matches to the LipidSearch library. A *p*-value < 0.05 indicates a significant effect. Abbreviations: T-statistic (T-stat) and log2 fold change (Log2FC). TG = triacyglyceride, PC = phosphatidylcholine, lysoPC = lysophosphatidylcholine, PE = phosphatidylethanolamine, CerG1 = monoglycosylceramide, Cer = ceramide. A positive Log2FC indicates a lower abundance in the MFGM-fed piglets and vice versa.

Lipid Species	T Stat	Log2FC (Control/High)	*p*-Value
TG 53:7; [M + NH4]+	4.03	0.80	0.001
TG 54:4; [M + NH4]+	3.95	0.65	0.002
TG 58:3; [M + NH4]+	3.66	0.75	0.003
TG(18:0/18:1/18:1) + NH4_MS2	3.55	0.65	0.004
TG(16:0/18:1/18:2) + NH4_MS2	3.46	0.63	0.004
TG 50:3; [M + NH4]+	3.41	0.70	0.005
TG 53:6; [M + NH4]+	3.34	0.67	0.005
TG(18:1/18:1/18:1) + NH4_MS2	3.32	0.51	0.005
TG(16:0/18:1/18:1) + _MS2	3.23	0.57	0.007
TG 48:2; [M + NH4]+	3.23	0.60	0.007
TG(16:0/18:1/18:1) + NH4_MS2	3.15	0.66	0.008
TG 58:2; [M + NH4]+	2.91	0.62	0.012
TG(18:1/14:0/18:1) + NH4_MS2	2.87	0.48	0.013
TG 57:2; [M + NH4]+	2.81	0.61	0.015
TG(18:0/18:1/18:1) + NH4_MS2	2.24	0.65	0.043
TG 58:1; [M + NH4]+	2.16	0.33	0.049
PC(40:5) + H_MS2	−2.62	−0.24	0.021
lysoPC 18:0; [M + H]+	−2.57	−0.22	0.023
plasmenyl-PE 34:0; [M + H]+	−2.56	−0.21	0.024
PC(36:1) + H_MS2	−2.52	−0.18	0.026
PE(18:0/18:1) + H_MS2	−2.51	−0.20	0.026
PC(36:1) + H_MS2a	−2.49	−1.23	0.027
CerG1(d38:1 + O) + _MS2	−2.36	−0.19	0.035
PC(36:2) + H_MS2	−2.35	−0.14	0.035
PC(34:0) + H_MS2	−2.34	−0.20	0.036
PC(40:4) + H_MS2	−2.27	−0.28	0.041
PE(18:0p/22:4) + H_MS2	−2.26	−0.19	0.042
Cer(d20:1/18:0) + H_MS2	−2.25	−0.35	0.042
PE(16:0p/22:6) + H_MS2	−2.18	−0.18	0.048
plasmenyl-PC 40:5; [M + H]+	−2.17	−0.23	0.049

## Data Availability

The data are available from the corresponding author upon reasonable request. The data are not publicly available due to data size and complexity of raw mass spectrometry files.

## Supplementary Materials

The following supporting information can be downloaded at: https://www.mdpi.com/article/10.3390/metabo12080689/s1, Figure S1: Boxplots of abundances of lipids detected as significantly different in the hippocampus of piglets fed a control of MFGM-supplemented infant formula. Figure S2 shows the PLS-DA loadings of hippocampus lipids detected in positive ionization mode. Figure S3 shows the Venn diagram of the number of lipid species found in the hippocampus positive ionization mode analysis that were key VIP features contributing to the significant PLS-DA model or found to be significant (*p*-value < 0.05, uncorrected for multiple testing) by *t*-test. The Supplementary tables.xslx file contains: Table S1 shows the annotated lipids found in piglet brains in both positive and negative ionization mode. Note, where annotations are followed by ‘_MS2’, the annotation was based on MS2 spectra using the LipidSearch tool., Table S2: Lipid class percentage composition of four piglet brain regions detected by positive and negative ionization modes (n = 23 animals). Table S3: *p*-values from correlation analysis of lipid classes from combined analysis of both ionization modes. A separate supplementary file (Supplementary Tables pvalues.xslx) of fold change and *p*-values provides the inter-tissue comparisons and the treatment comparisons within tissue.
