# Supplementary material for: Impacts of Formula Supplemented with Milk Fat Globule Membrane on the Neurolipidome of Brain Regions of Piglets
Source: Metabolites. 2022 Jul 26;12(8):689. doi: 10.3390/metabo12080689 (PMC9330244; doi:10.3390/metabo12080689)
Supplement: Supplementary file 1 [file metabolites-12-00689-s001.zip › metabolites-1799800-supplementary figures.pdf]

Supplementary figure file for: **Impacts of formula supplemented with milk fat globule membrane on the neurolipidome of brain regions of piglets**

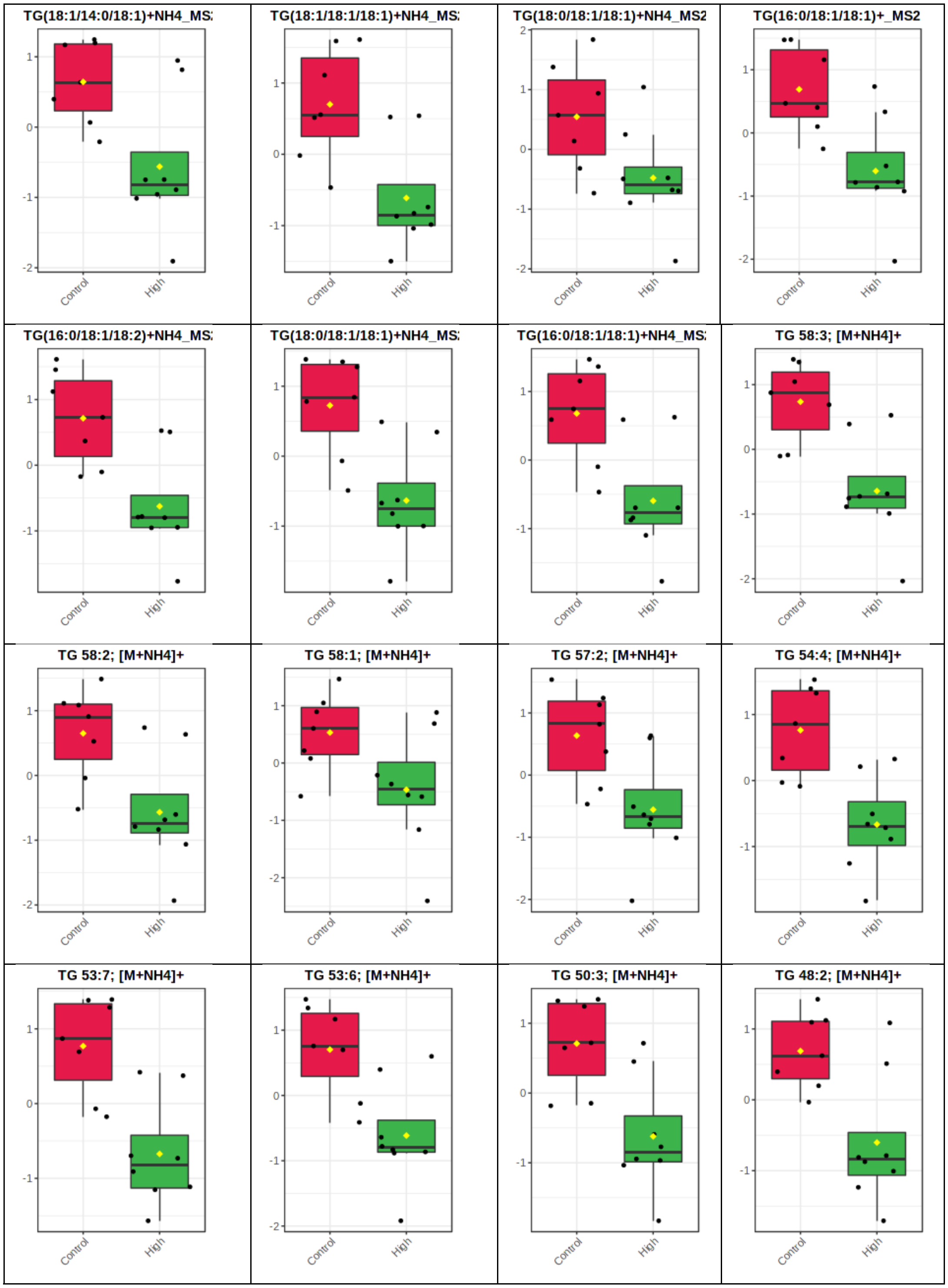

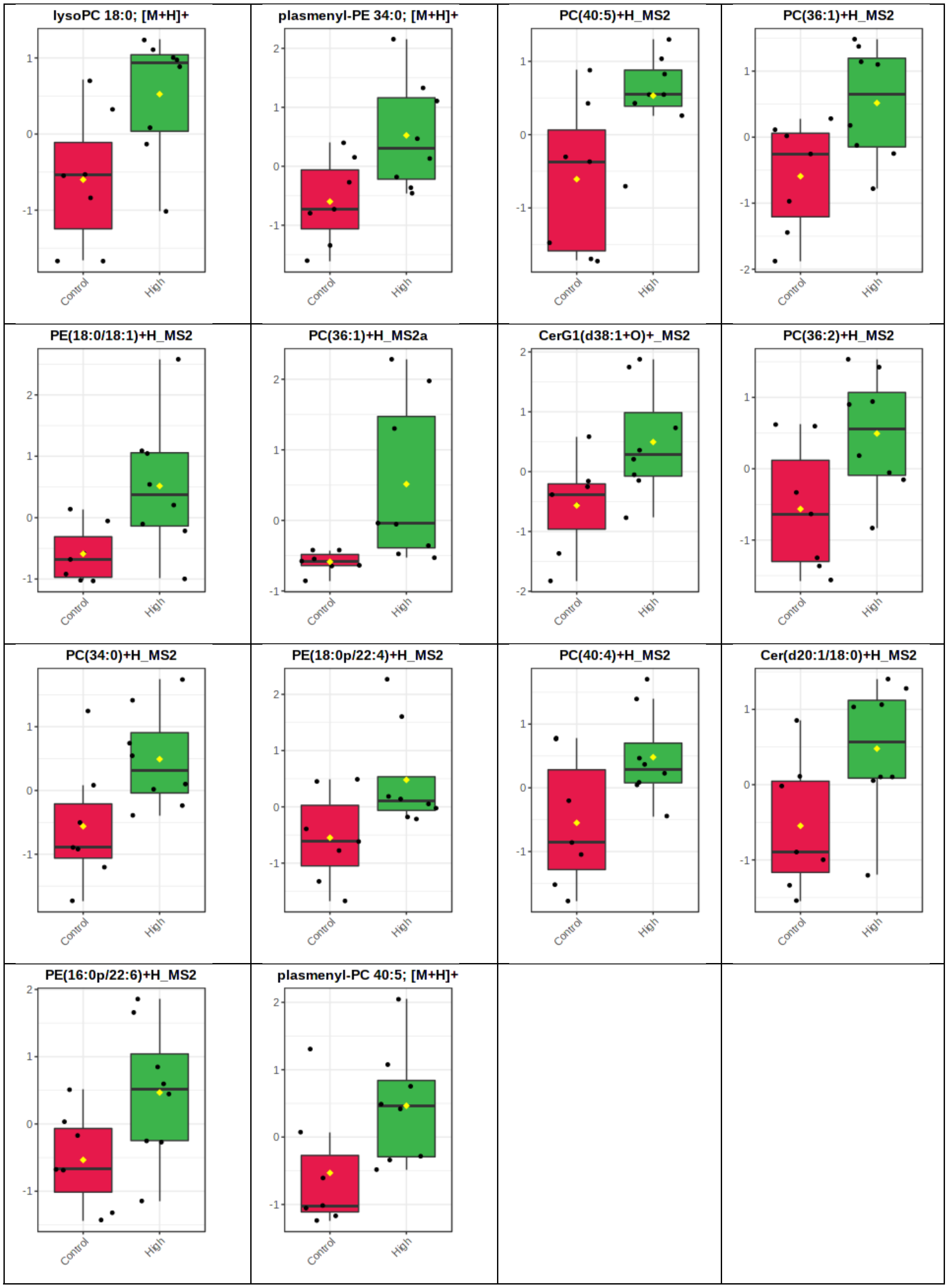

Supplementary Figure S1: Boxplots of abundances of lipids detected as significantly different in the hippocampus of piglets fed a control of MFGM supplemented diet.

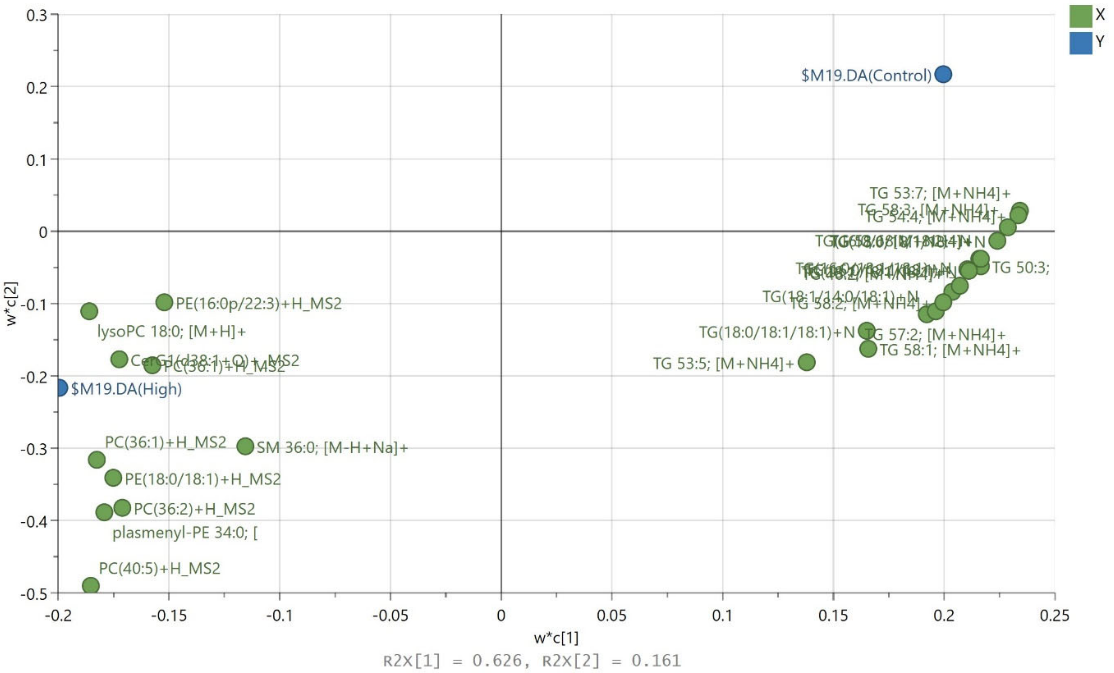

Supplementary Figure S2: PLS-DA loadings of hippocampus lipids detected in positive ionization mode.

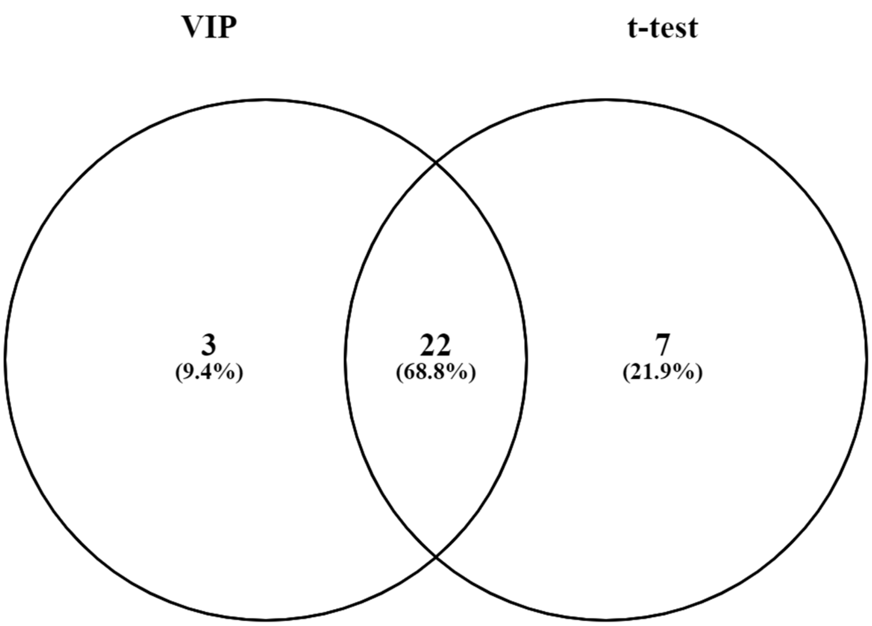

Supplementary Figure S3: Venn diagram of the number of lipid species found in the hippocampus positive ionization mode analysis that were key VIP features contributing to the significant PLS-DA model or found to be significant (p-value <0.05, uncorrected for multiple testing) by t-test.
